# REMOD: A Tool for Analyzing and Remodeling the Dendritic Architecture of Neural Cells

**DOI:** 10.3389/fnana.2015.00156

**Published:** 2016-01-06

**Authors:** Panagiotis Bozelos, Stefanos S. Stefanou, Georgios Bouloukakis, Constantinos Melachrinos, Panayiota Poirazi

**Affiliations:** ^1^Institute of Molecular Biology and Biotechnology (IMBB), Foundation for Research and Technology-Hellas (FORTH)Crete, Greece; ^2^Department of Molecular Biology and Genetics, Democritus University of ThraceCrete, Greece; ^3^Department of Biology, University of CreteCrete, Greece; ^4^Computer Science Department, University of CreteCrete, Greece

**Keywords:** neuron, dendrite, dendritic remodeling, computational tool, statistical analysis

## Abstract

Dendritic morphology is a key determinant of how individual neurons acquire a unique signal processing profile. The highly branched dendritic structure that originates from the cell body, explores the surrounding 3D space in a fractal-like manner, until it reaches a certain amount of complexity. Its shape undergoes significant alterations under various physiological or neuropathological conditions. Yet, despite the profound effect that these alterations can have on neuronal function, the causal relationship between the two remains largely elusive. The lack of a systematic approach for remodeling neural cells and their dendritic trees is a key limitation that contributes to this problem. Such causal relationships can be inferred via the use of large-scale neuronal models whereby the anatomical plasticity of neurons is accounted for, in order to enhance their biological relevance and hence their predictive performance. To facilitate this effort, we developed a computational tool named REMOD that allows the structural remodeling of any type of virtual neuron. REMOD is written in Python and can be accessed through a dedicated web interface that guides the user through various options to manipulate selected neuronal morphologies. REMOD can also be used to extract meaningful morphology statistics for one or multiple reconstructions, including features such as sholl analysis, total dendritic length and area, path length to the soma, centrifugal branch order, diameter tapering and more. As such, the tool can be used both for the analysis and/or the remodeling of neuronal morphologies of any type.

## Introduction

The morphological complexity of dendrites has been documented since the times of Ramón y Cajal (1911) and is generally considered as an important factor for the proper functioning of the nervous system. Dendritic morphology also demonstrates a great deal of variation across different neuronal cell types (Ascoli, 2006), thus imposing an extra layer of complexity onto the deeply tangled relationship between structure and function (Mainen and Sejnowski, 1996; Krichmar et al., 2002; Schaefer et al., 2003). This structural diversity has been suggested to play a key role in shaping the mode of connectivity between neurons (Sholl, 1956; Kalisman et al., 2003; Chklovskii, 2004) as well as the information processing and signal integration capabilities of neural cells (reviewed in Spruston, 2008). Hence, the morphology of the dendritic tree directly influences the functionality of neural tissue, both at the single-cell and network levels, in complex ways.

To deliver this structural intrication neurons appear to follow an innate growth program (McAllister, 2000; Libersat and Duch, 2004) while responding adaptively to extrinsic guidance cues supplied by the extracellular matrix (for detailed reviews, see Wong and Wong, 2000; Procko and Shaham, 2010). Moreover, calcium signaling events induced by the electrophysiological activity of neurons are regarded as indispensable part of the morphogenesis process (Wu and Cline, 1998; Wong and Ghosh, 2002; Lohmann et al., 2005). Importantly, dendrites appear to be dynamic structures, even after the post-developmental period (Magariños et al., 1996; Stranahan et al., 2007) and these morphological changes ought to be related to neuronal function.

A considerable amount of research has accumulated to validate this notion, by quantifying the dendritic alterations that happen in response to either physiological or neuropathological stimuli. For instance, in Alzheimer’s disease, besides the well-known histopathological hallmarks of extracellular amyloid plaques and intracellular neurofibrillary tangles (NFTs), dendritic atrophy is consistently found among hippocampal and cortical pyramidal neurons (Yamada et al., 1988; Moolman et al., 2004). In another study, when rats were reared under chronic stress conditions, abnormal morphological changes to dendritic trees were seen across at least three different brain regions: the hippocampus, amygdala and prefrontal cortex (Vyas et al., 2002; Shansky and Morrison, 2009). Interestingly, neurons in these areas seemed to respond completely different to the same type of stress. In the CA3 subregion of the hippocampus, as well as in the medial prefrontal cortex, the dendritic arbor retracted to itself. On the contrary, dendritic trees in the basolateral amygdala (BLA) exhibited excessive growth as evident by their increased total length and number of branches.

Dendritic remodeling is also happening under physiological conditions. For instance, when there is increased need for receiving, integrating and encoding complex spatiotemporal patterns about newly encountered environments, the dendritic arbors of rat hippocampal neurons grow (Tronel et al., 2010); perhaps to accommodate new synapses and/or counterbalance the impending overexcitability, that otherwise would be toxic to the cell. Despite the profound effect that such dendritic alterations can have on neural processing, a causal relationship between structural changes and neuronal output has not been established yet.

A great deal of neuroanatomical research effort has been devoted to providing a direct link between structure and function, at both the single-cell and network levels. During the past few decades, the advent of intracellular labeling techniques and the application of various visualization methods have led to a dramatic increase in high resolution dendritic morphology data. A tremendous progress in imaging methods and automation is also expected to pave the way for an exponential growth of the data acquisition in the forthcoming years (Peng et al., 2015). The acquired morphological reconstructions are stored in dedicated databases such as the NeuroMorpho (Ascoli et al., 2007), the Fly Circuit (Chiang et al., 2011) and the Cell-Centered Database (Martone et al., 2003), and enable the quantitative analysis of neuronal shapes by the use of parameters relevant to the metrical and topological properties of the cell. Investigators interested in deciphering the role of neuroanatomy to the proper functioning of the nervous system have developed and provided the community with commercial and freeware tools such as Neurolucida Explorer (Glaser and Glaser, 1990), L-Measure (Scorcioni et al., 2008) BTMORPH (Torben-Nielsen, 2014), and NLMorphologyViewer^1^ to trace, analyze and visualize the shape of the reconstructed neurons. However, most of these tools need to be locally installed and often require at least some basic programming skills, making them difficult to use by non-experts (for a detailed review, see Parekh and Ascoli, 2013).

More advanced approaches to the remodeling of dendritic trees incorporate the de-novo generation of neuronal morphologies, based either on intrinsic correlations between morphometric parameters (e.g., the NETMORPH tool by Koene et al., 2009), on principles of neuronal material conservation vs. conduction times (e.g., the TREES toolbox by Cuntz et al., 2011), or the dynamic and competitive interplay of retraction and outgrowth processes (e.g., the CX3D tool by Zubler et al., 2013; Hjorth et al., 2014). Others implement a hybrid of local and global generative algorithms to efficiently reproduce the anatomical variability of various neuronal classes (e.g., the L-Neuron and ArborVitae tools by Ascoli et al., 2001). Recently, the NeuroMac tool introduced an interesting context-aware approach in which developing neurons interact with the surrounding brain substrate (Torben-Nielsen and De Schutter, 2014). On the core of all the above algorithms lays the use of a limited set of statistical descriptors and assumptions on growth rules to generate stochastic neuronal structures that are statistically indistinguishable from the real neurons of the same morphological class. There is little evidence as to whether these assumptions and rules would hold under different physiological and/or pathological conditions. While the importance of these tools is widely recognized by the neuroanatomical community, to our knowledge, there is currently no tool available to implement targeted, assumption-free alterations on specific dendrites or branches of already-grown morphologies. Given that dendritic remodeling is happening quite frequently in nature, as part of the ever-adapting neuronal function, the necessity of a tool to remodel the dendritic morphology of digital reconstructions is long overdue. Moreover, the recent use of detailed biophysical and morphologically realistic large-scale brain models to investigate microcircuit structure and function (Markram et al., 2015) highlights the need to understand how different anatomical features and their plasticity shape brain function and dysfunction. Towards this goal, a systematic methodology that allows efficient remodeling of any type of neuronal morphology is a prerequisite.

In this work, we introduce REMOD, a computational tool that allows the structural remodeling of any type of digitally reconstructed neuron. The algorithm focuses on the simulation of the end-result of a dendritic remodeling process, without explicitly implementing any growth and/or retraction rules. It should be noted that such an end result can be achieved by several different sets of manipulations, some of which may not be biologically relevant. The important advantage of REMOD is that it provides the flexibility to choose which manipulations are considered relevant for each experimenter, condition and cell type, thus allowing a full exploration of the parameter space. This flexibility is particularly important when investigating the effects of specific morphological manipulations on a given response pattern. Such manipulations can be used to tease out the contribution of distinct morphological factors from other processes such as biophysical mechanisms.

The tool is written in Python and can be accessed through a dedicated web interface that guides the user through various options to manipulate selected neuronal morphologies. More explicitly, it provides the ability to upload one or more morphology files (in SWC, the most widely used format) and choose specific dendrites or dendritic regions on which to operate one of the following actions: shrink, remove, extend, branch or scale. The user retains complete control over the extent of each alteration and if a chosen action is not possible due to pre-existing structural constraints, appropriate warnings are generated. It is worth mentioning that REMOD can also be used to extract a plethora of descriptive statistics for one or multiple neurons, such as sholl analysis, total dendritic length and area, path length to the soma, centrifugal branch order, diameter tapering and more. As such, the tool can be used both for the analysis and/or the remodeling of neuronal morphologies of any type.

## Methods

Recent advances in high-throughput single-neuron imaging techniques are expected to stimulate a morphological data “explosion” that will revolutionize the computational neuroanatomy field. Importantly, the aforementioned data “explosion” needs to be accompanied by the development of software tools, designed to meet the future requirements of parsing and manipulating tons of 3D neuron reconstruction data in a transparent, reliable and highly-consistent manner (Peng et al., 2015). In order to achieve this longer-term goal, an appropriate approach needs to be adopted by the community to properly address this “big data” challenge. Therefore, it is imperative for the new generation of software packages to be optimized for this data volume, to handle it in a user friendly way and with the added benefit to be open-source and publicly available, allowing ease of use, sharing and active development by avid researchers in the field.

The proposed tool is developed according to this philosophy. The software implementation is in Python 2.7 and the tool is open source and available for use as a web-application at www.remod.gr/. The source code and documentation of the tool is available on the Github repository hosting service at github.com/bozelosp/remod, where it can be downloaded as a standalone application that retains almost all of its online capabilities and/or further developed.

The software package contains flexible and extendable modular blocks of code that can be generally classified in three categories: (i) parsing the morphology reconstruction data encoded in standard SWC files; (ii) performing morphometric and statistical analysis of the provided dendritic trees; and (iii) executing specific remodeling actions and exporting the new morphologies in the SWC format. The following paragraphs explain the user-action workflow between the abovementioned categories.

**Figure 1:**
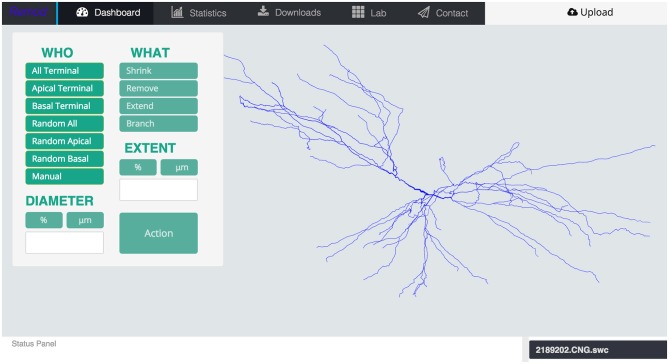
**Screen shot of the main page of the tool depicting an uploaded morphology reconstruction of a CA3 pyramidal neuron**.

First, the user uploads one or more morphology reconstructions, properly formatted as SWC files. REMOD parses the geometric information of the specific 3D structure and topology and provides a rotating 3D visualization of the corresponding tree, as well as many depictions of the extracted morphometric statistics in the form of tables and charts. Next, the interface guides the user through a range of remodeling options that can be implemented on the selected morphologies as shown in Figure 1.

For example, the researcher can manually select specific dendrites to impose a morphological alteration or more conveniently select a whole region of the neuron (i.e., the basal or apical tree), or even a randomly selected portion of it. Remodeling actions can be implemented on any and/or multiple selected dendrites, irrespectively of whether they are terminal branches or not.

**Figure 2:**
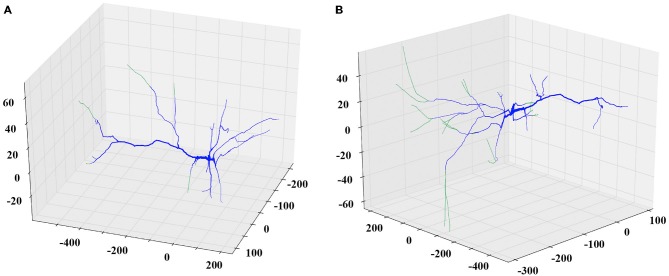
**Examples of the extending and branching REMOD actions on a PFC pyramidal neuron.** The induced morphological changes (added segments) are shown in green. **(A)** All apical terminal dendrites are extended by 50% of their initial length. **(B)** New branches are added to the tip of the stem of all basal terminal dendrites. The produced length of each one of the branches is 80% of the parent length.

Next, the researcher decides the remodeling action to apply. If the plan is to further grow the dendritic tree, then the appropriate options to choose might be additive extension or branching, i.e., adding two new dendrites stemming from an existing parental one. The researcher can specify the extent of growth in terms of a percentage of the previous dendritic length or by defining the desirable length in micrometers. In this case, the algorithm will generate a series of somewhat randomly directed cylinders that radiate away from the parental dendritic segment and the soma as shown in Figure 2. The added segments have random lengths sampled from a realistic distribution and mimic the way dendrites explore the available 3D space in a quasi-random, quasi-directed way.

**Figure 3:**
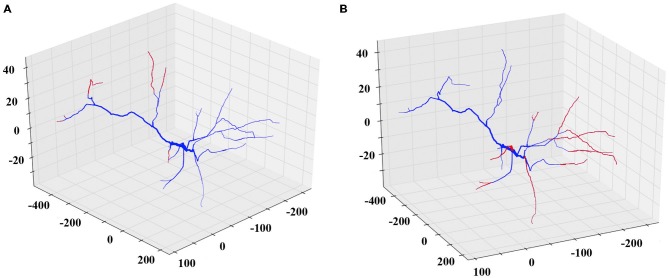
**Examples of the shrinking and removing REMOD actions on a PFC pyramidal neuron.** The induced morphological changes (removed segments) are shown in red. **(A)** All apical terminal dendrites are shrunk by 60% of their initial dendritic length. **(B)** All basal terminal dendrites are completely removed.

Similarly, to simulate a dendritic retraction, the user decides between two options: shrinking or complete removal of the selected dendrites. In both actions, cylinders are removed from the SWC file to match the desirable reduction in length, without altering any other parts of the morphology file. An example of each case is shown in Figure 3.

**Figure 4:**
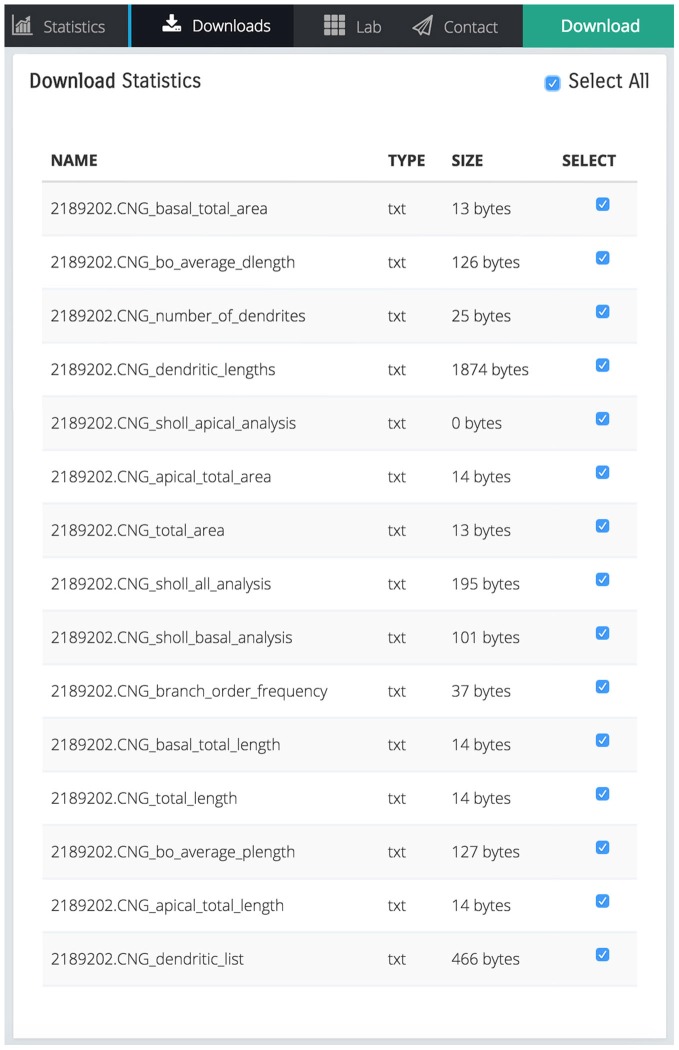
**Screen shot illustrating a list of downloadable files that contain various statistics calculated by REMOD for the uploaded CA3 pyramidal neuron depicted in Figure 1**.

The scaling action can be used to enlarge or reduce the dimensions of either the entire dendritic tree, or the basal/apical regions of the tree, independently. Remodeled morphologies are exported in the SWC standard format and can be downloaded and/or sent to the user via email as shown in Figure 4.

**Figure 5:**
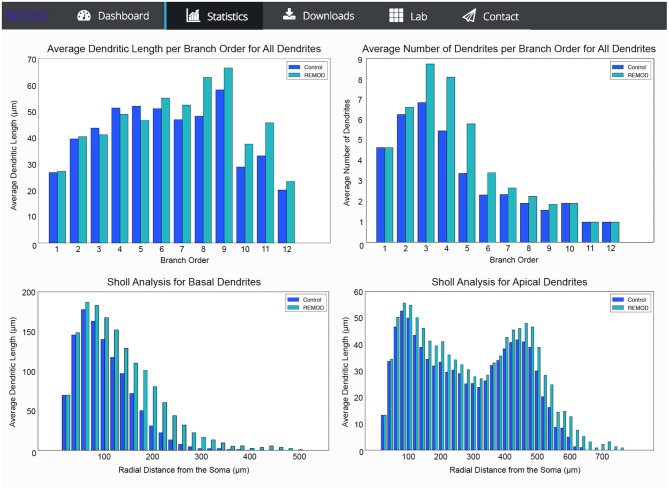
**Screen shots illustrating various comparison diagrams for a control group of 103 neocortical pyramidal neurons that has been remodeled by the use of the following REMOD actions: 40 percent of the basal terminal dendrites were selected for branching at 50% of their length and thereafter a 50% of the apical terminal dendrites were selected for extension by 60% of their parent length**.

REMOD is also able to extract useful statistics for the morphological and topological features of the neuronal reconstructions. Metrics such as total dendritic length, path length, surface and volume are available for both the basal and the apical regions of the tree. Sholl analysis is implemented either for the number of branch points, the number of intersections or the sum of dendritic lengths included in defined radius steps from the soma. The tool also supports the comparison between two groups of morphologically different neurons using the abovementioned metrics, as shown in Figure 5. This utility can be especially useful for both experimentalists and modelers wishing to identify anatomical differences between two particular groups of neurons.

## Results

To demonstrate the capabilities of REMOD we implemented morphological changes that led to dendritic atrophy and enhanced dendritic arborization, in hippocampal and amygdaloid pyramidal neurons, respectively. Such changes were reported to occur as a result of stress by Vyas et al. (2002). The modifications performed aimed at reproducing the percentage of change in various dendritic features as reported experimentally and were applied to a large number of morphologies downloaded from the NeuroMorpho database.

**Figure 6:**
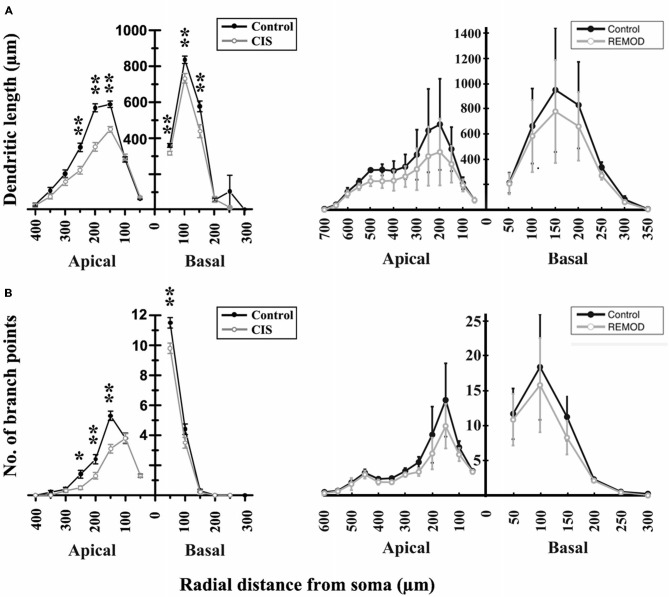
**Comparison of the experimentally induced (CIS) shrinkage in dendritic structure of the CA3 cells (control cells, *n* = 50; CIS cells, *n* = 45) with the same remodeling effect induced by REMOD on 25 CA3 neuronal reconstructions downloaded from NeuroMorpho database. (A)** Side by side comparison of the diagram showing the dendritic length as a function of radial distance from the soma as taken from Vyas et al. (2002, left), with the corresponding diagram of the remodeling example using REMOD (right). Changes in apical and basal dendrites are shown separately. **(B)** Comparison of the diagram showing the number of branch points as a function of radial distance from the soma as taken from the paper published by Vyas et al. (2002, left), with the corresponding diagram using REMOD.

According to Vyas et al. (2002) chronic immobilization stress (CIS) led to a significant decrease of the total dendritic length and the number of branch points in hippocampal CA3 pyramidal neurons as compared with neurons of the same class in control animals. The marked shrinkage was evident in both apical as well as basal dendrites of the examined pyramidal cells. Specifically, the total dendritic length of apical dendrites was decreased by 29%, and the number of apical branch points was reduced by 31%. The same analysis applied to basal dendrites found their total dendritic length shrunk by 16% and their number of branch points reduced by 16%, respectively. Based on the observation that the number of branch points is affected, it’s easy to deduce that a simple scaling of the entire dendritic tree length would not reproduce the observed phenotype. With the goal of simulating the overall shrinkage effect and reproducing the reported percentages, we took the following approach:

- Twenty five reconstructed neuronal morphologies of the hippocampal CA3 pyramidal class were obtained from the NeuroMorpho repository.
- Subsequently, the parameters that were used to remodel the morphologies were selected in such a way as to simulate the experimentally observed results. The total dendritic length of the apical tree was reduced by randomly selecting 17% of the terminal dendrites for complete removal and thereafter randomly choosing an additional 10% of the remaining terminal dendrites to shrink by 18%.
- Accordingly, the basal tree was pruned by randomly selecting 15% of its terminal dendrites for complete removal and shrinking another 5% of them by 5% of their initial length. The results of this processing are shown in Tables 1, 2 and Figure 6.

**Table 1:** **Difference in total dendritic length of CA3 pyramidal neurons as reported experimentally after CIS and via remodeling with REMOD**.

Total dendritic length	Control (experiment)	CIS	% Change	Control (NeuroMorpho)	Remodeled	% Change
Apical	2113	1498	−29	5471	3922	−28.31
Basal	1827	1527	−16	4302	3623	−15.78

**Table 2:** **Difference in the number of branch points of CA3 pyramidal neurons as reported experimentally after CIS and via remodeling with REMOD**.

Number of branch points	Control (experiment)	CIS	% Change	Control (NeuroMorpho)	Remodeled	% Change
Apical	14.7	10.2	−31	31.14	21.18	−31.98
Basal	16.2	13.6	−16	29.22	24.62	−15.74

It should be noted that other combinations of dendrite removal/pruning may also reproduce the reported averaged changes in dendritic length. Due to the lack of information as to which specific morphological features were altered in the experiments, we used a randomized approach.

**Figure 7:**
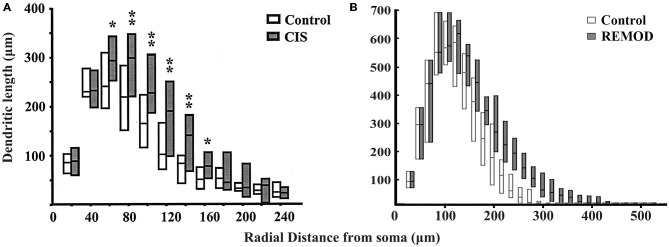
**Comparison of the experimentally induced (CIS) increase in dendritic arborization of the basolateral amygdala (BLA) cells (control cells, *n* = 18; CIS cells, *n* = 22) with the same remodeling effect induced by REMOD on 26 BLA neuronal reconstructions downloaded from NeuroMorpho database.** Side by side comparison of the diagram showing the dendritic length as a function of radial distance from the soma as taken from the paper published by Vyas et al. (2002) **(A)** with the corresponding diagram of the remodeling example using REMOD **(B)**.

Interestingly, the same CIS paradigm that caused dendritic atrophy in CA3 pyramidal neurons induced the opposite effect in BLA pyramidal neurons. The total dendritic length of BLA pyramidal neurons under conditions of CIS increased by 25% and the number of branch points by 15% compared to the control cells. With the goal of reproducing the experimentally obtained results of dendritic extension in the amygdala we implemented the following series of actions in REMOD:

- Twenty six reconstructed neuronal morphologies of the BLA pyramidal class of neurons were obtained from the NeuroMorpho repository.
- Subsequently, 10 percent of the terminal dendrites were selected for branching at 80 percent of their length and thereafter a 20% of all the available terminal dendrites were selected for extension by 70% of their initial length. The results are shown in Tables 3, 3, 4.
- To further our analysis, we performed segmental processing to track the changes in dendritic length and number of branch points as functions of radial distance from the cell soma. The results are shown in Figure 7.

**Table 3:** **Difference in the total dendritic length of BLA pyramidal neurons as reported experimentally after CIS and via remodeling with REMOD**.

Total dendritic length	Control (experiment)	CIS	% Change	Control (NeuroMorpho)	Remodeled	% Change
All	1330	1666	25	4040	5067	25.42

**Table 4:** **Difference in the number of branch points of BLA pyramidal neurons as reported experimentally after CIS and via remodeling with REMOD**.

Number of branch points	Control (experiment)	CIS	% Change	Control (NeuroMorpho)	Remodeled	% Change
All	13.5	15.0	11	25.98	28.825	10.95

Overall, despite the fact that the reconstructed morphologies analyzed by Vyas et al. (2002) were significantly smaller than the neurons of the same class downloaded from the NeuroMorpho database, we reliably reproduced the percentage morphometric changes as well as the overall distributions of changes between the control and CIS-treated cells in both neuronal classes.

## Discussion

Over the past decade, computational simulations of the electrophysiological behavior of neurons have become fairly common as neuroscientists strive to develop a comprehensive understanding of the nervous system (Bower, 2013; Dudai and Kathinka, 2014). At the same time, the rapid advancement of imaging techniques has greatly enhanced the acquisition of high-resolution 3D neuronal reconstructions, resulting in anatomically precise and biophysically detailed compartmental models that render realistic simulation of neuronal behavior possible under virtual experimental conditions. Thus, it is expected that multi-compartmental cable models with ever-increasing accuracy in detail will become critically important to better understand the rich repertoire of computational operations performed by dendrites.

In addition, substantial progress has been achieved by experimental neuroscientists into revealing how neural plasticity might relate to development, learning and disease and how its influence could be projected far beyond the dynamics of synapses (Butz et al., 2009; Yau et al., 2011). Along with motile spines and axonal branches, the dendritic architecture of neural cells is also subject to significant morphological readjustments (for a detailed review, see Emoto, 2011). Thus, many challenging questions arise about the structural plasticity of the nervous system, including the rules that govern the dendritic morphological configuration, the adaptability of the neural connectivity and the preservation of functional continuity. Hence, a software tool that is able to induce structural alterations upon virtual neurons and statistically quantify the changes between acquired and remodeled morphologies could prove an invaluable resource for modelers and neuroanatomists alike, complementary to the existing approaches.

In this paper, we have presented REMOD, a novel open-access software tool for the analysis and remodeling of dendritic morphologies. The tool is written in the non-commercial Python programming language and is publicly available on the GitHub platform, under the open-source MIT license to encourage active development by the neuroanatomical community. The tool is accompanied by an easily accessible front-end interface that anyone can use via a web browser (http://www.remod.gr), thus eliminating the need to download and configure additional packages and/or restricted libraries locally on the desktop.

REMOD is designed to fulfill the emerging need among computational neuroscientists to simulate the dynamic nature of the dendritic structure. Other toolboxes have been developed for the special purposes of de novo generating synthetic neurons or neuronal networks, or even configuring the connectivity between them (Kalisman et al., 2003; Koene et al., 2009). These methods are exploiting experimentally-substantiated principles, such as the conservation of neuronal material vs. conduction time constraints, the dynamic negotiation between retraction and outgrowth processes and the context-awareness of neuronal arborization, in order to enforce local or global generative algorithms that efficiently replicate the extent of morphological variability manifested by various neuronal types (Ascoli et al., 2001; Cuntz et al., 2011; Zubler et al., 2013; Hjorth et al., 2014; Torben-Nielsen and De Schutter, 2014). Still, the modeling of structural plasticity in the brain is arguably hindered by a multitude of challenges. These challenges predominantly include the complex interaction between numerous interdependent factors, such as genetic, metabolic and behavioral influences, that cannot be easily dissected for integration into the current predictive models (Bestman Da Silva and Cline, 2008; Polleux and Ghosh, 2008).

To overcome this limitation, the researcher can approach morphology from a different methodological perspective, that of remodeling an already-existing neuronal structure ad arbitrium. Principally, this approach provides a wider flexibility to choose the structural manipulations that are considered more relevant for each experimenter, physiological condition and cell type, albeit allowing for a greater exploration of the available parameter space. This type of freedom could prove particularly important when investigating the effects of certain morphological alterations on a given neuronal response pattern, even if they are unlikely to occur. Such manipulations can for instance be used to assess and tease out the individual contribution of distinct morphological factors from other processes, like the electrophysiological properties of ion channels. Furthermore, REMOD offers the ability to perform statistical morphometric analyses of the user-provided morphologies via uploading to our server, an additional utility that may also be of great service to experimentalists.

In this work, we sought to demonstrate the capabilities of our toolbox by reproducing dendritic remodeling in the hippocampus and the amygdala brain regions of the rat under stress conditions, as reported by Vyas et al. (2002). Using REMOD, we faithfully reproduced the percentage of morphological changes reported in this paper, by imposing dendritic remodeling to 3D reconstructions of the same neuronal types downloaded from the NeuroMorpho repository. Unfortunately we did not have access to the original morphologies in order to perform a direct comparison.

The reproduction of the experimental results (with respect to the percentage of changes observed), delivered using REMOD, reflects the ability of the tool to simulate complicated neuronal phenomena that may occur under physiological or pathological conditions. We believe that the functions provided within the first release of the tool are flexible and efficient enough to simulate any type of dendritic remodeling, capturing the variant expression of neuronal adaptability that has been extensively documented, but still insufficiently explained in current theoretical models.

To the best of our knowledge, REMOD is the first software package that allows the remodeling of existing, already-grown, detailed neuronal morphologies, in parallel with the effortless extraction of morphological descriptive statistics. Thus, REMOD allows the implementation of a systematic approach for altering virtual neuronal morphologies, which is likely to promote further research into understanding the hidden associations between critical neuroanatomical characteristics and the distinct electrophysiological patterns that individual neurons, as well as neural networks, exhibit. Exploiting the benefits and the capabilities of dendritic remodeling will aid the transition from investigating a “rigid” neuronal function to a refined exploration of the intricate effect of morphology to dendritic function and neuronal processing.

## Conflict of Interest Statement

The authors declare that the research was conducted in the absence of any commercial or financial relationships that could be construed as a potential conflict of interest.

## Footnotes

^1^http://neuronland.org/NLMorphologyViewer/NLMorphologyViewer.html
